# Calibration Methods for Time-to-Digital Converters †

**DOI:** 10.3390/s23052791

**Published:** 2023-03-03

**Authors:** Wassim Khaddour, Wilfried Uhring, Foudil Dadouche, Norbert Dumas, Morgan Madec

**Affiliations:** ICube Research Institute, University of Strasbourg, CNRS, UMR 7357, 23 Rue du Loess, CEDEX, 67037 Strasbourg, France

**Keywords:** asynchronous TDC, average-bin-width calibration, bin-by-bin calibration, calibration techniques, differential non-linearity (DNL), integral non-linearity (INL), matrix calibration, synchronous TDC, time-to-digital converter (TDC)

## Abstract

In this paper, two of the most common calibration methods of synchronous TDCs, which are the bin-by-bin calibration and the average-bin-width calibration, are first presented and compared. Then, an innovative new robust calibration method for asynchronous TDCs is proposed and evaluated. Simulation results showed that: (i) For a synchronous TDC, the bin-by-bin calibration, applied to a histogram, does not improve the TDC’s differential non-linearity (DNL); nevertheless, it improves its Integral Non-Linearity (INL), whereas the average-bin-width calibration significantly improves both the DNL and the INL. (ii) For an asynchronous TDC, the DNL can be improved up to 10 times by applying the bin–by-bin calibration, whereas the proposed method is almost independent of the non-linearity of the TDC and can improve the DNL up to 100 times. The simulation results were confirmed by experiments carried out using real TDCs implemented on a Cyclone V SoC-FPGA. For an asynchronous TDC, the proposed calibration method is 10 times better than the bin-by-bin method in terms of the DNL improvement.

## 1. Introduction

The role of a TDC is to measure precise time intervals between two events represented by two signals (reference and measured signals) [1,2,3,4], which is the keystone of many applications such as LIDAR applications [5], time-resolved fluorescence measurement [6], fluorescence lifetime imaging [7], 3-D active imaging and time-correlated photon counting [8]. In general, high-resolution TDCs can be built as Application-Specific Integrated Circuits (ASICs) [9]. However, for many applications, it can be better to implement the TDC on field-programmable gate arrays (FPGAs) due to the flexibility and reconfigurability, as well as the short development time of these circuits [10,11,12]. Moreover, the integration of hard processor systems in System-on-Chip FPGA (SoC-FPGA) kits allows performing an on-chip downstream processing such as a post-calibration process [11].

The Coarse–Fine architecture is extensively used to build FPGA-based TDCs to provide a high resolution and a large dynamic range [13,14,15]. The coarse TDC defines the TDC’s dynamic range; it is generally a classical counter clocked by the global system’s clock, whereas the fine TDC, which defines the resolution, is commonly based on the time interpolation technique [16]. The most common structure to build an FPGA-based fine TDC is the Tapped Delay Line (TDL) [14,17]. TDCs can be classified into two main categories: synchronous and asynchronous TDCs. In the synchronous TDC, the reference signal is synchronous with the system clock, and this type of TDCs consists of a coarse and a fine TDC. In contrast, in asynchronous TDCs, both the reference and the measured signals are asynchronous with the clock; thus, these TDCs include a coarse and two fine TDCs, for example, double-TDL. Generally, TDL-based TDCs implemented in FPGA suffer from a large non-linearity due to the large variations in the delay of the TDL cells [18]. This crucial drawback imposes calibrating the TDC to improve the linearity. The most prevailing calibration methods are the average-bin-width and the bin-by-bin methods [1,19,20]. In recent years, many studies have addressed these two methods, especially the bin-by-bin calibration. Nevertheless, most of these works have used this method for the calibration of individual time interval measurement, such as single-shot measurement and averaged measurement, without covering the calibration of histograms, which is an essential procedure for many TDC-based applications. In previous works [21,22,23], time measurement standard deviations of about 18 ps (0.4 LSB), 12.2 ps (1.07 LSB) and 9 ps (0.34 LSB) were achieved, respectively, through bin-by-bin calibration. However, these works did not apply this calibration method to histogram measurements. Furthermore, most of the studies about TDC calibration have predominantly focused on synchronous TDCs [24,25], and very few of them have discussed asynchronous TDCs. The aim of this paper is twofold: (i) to present the methodology and to compare the performance of the aforementioned methods when applied to histograms in the case of synchronous TDCs, and (ii) to propose an innovative robust calibration method for asynchronous TDCs named the “Matrix calibration”. This method, previously presented in [26], is performed as a post-processing of raw data for either ASIC- or FPGA-based TDCs. For its evaluation, this method is compared to the bin-by-bin method which is usually adopted for the calibration of asynchronous TDCs [27].

This paper starts with an introduction about Coarse–Fine TDCs. Section 2 covers the general functional principle of a Coarse–Fine synchronous TDC as well as its most used calibration methods. Section 3 firstly introduces the operating principal and calibration methods of asynchronous TDCs, then presents our proposed calibration method named the “Matrix calibration” in depth. The simulation and experimental results for the calibration of synchronous and asynchronous TDCs are, respectively, presented in Section 4 and Section 5. Lastly, Section 6 provides a brief conclusion of this work.

## 2. Synchronous TDCs Calibration

This section introduces the operating principle of synchronous TDCs. Then, it discusses the two reference methods of calibration of this type of TDCs, which are the bin-by-bin and the average-bin-width calibration methods.

### 2.1. Operating Principle of Synchronous TDCs

**Figure 1:**
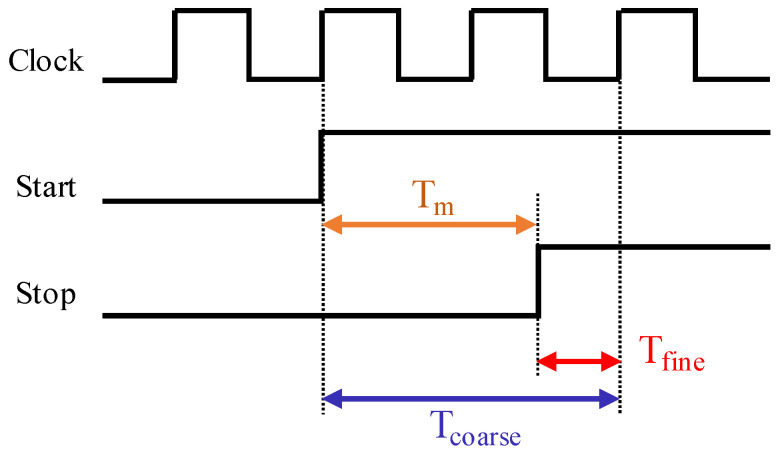
Synchronous Coarse–Fine TDC principle. T_m_ can be measured as a subtraction of two components, T_coarse_ and T_fine_.

A TDC is an essential device for the measurement of precise time intervals between two events represented by two signals called “Start” and “Stop”. For synchronous Coarse–Fine TDCs, the Start signal is synchronized to the TDC system clock. Thus, the measured time interval Tm can be measured as a subtraction of two components: T_coarse_, which is the number of system clock cycles between the Start signal and the first clock’s rising edge after the Stop signal multiplied by the clock period, and T_fine_, which is the interval from the Stop rising edge to the next clock rising edge, as illustrated in Equation (1) and Figure 1.

T_m_ = T_coarse_ − T_fine_ (1)

Hence, a synchronous Coarse–Fine TDC should contain two parts: a counter running at the system clock to measure T_coarse_ and a fine block, which is usually a time interpolation structure, to measure T_fine_.

### 2.2. Calibration Methods for Synchronous TDCs

**Figure 2:**
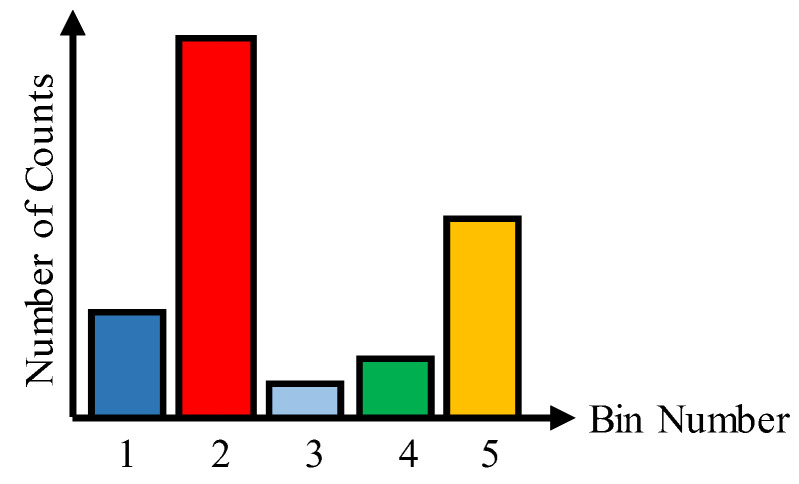
Code density histogram of a simple synchronous TDC with five bins.

The most popular calibration methods for synchronous TDCs are the bin-by-bin and the average-bin-width calibration methods. These methods require determining the raw bins’ widths by performing a code density test. The code density histogram integrates a sufficient number of counts in a way that the Stop signal arrives with random delays that cover the full range of the TDC. Thus, in the resulting histogram, each bin contains a number of counts proportional to its width. To illustrate the purpose, let us consider a five-bin TDC with a total delay of T. Figure 2 presents a code density histogram of this simple TDC. The mentioned methods are discussed as follows:

#### 2.2.1. Bin-by-Bin Calibration

This method readdresses the TDC raw bins to calibrated times or calibrated bins by means of a lookup table (LUT). A code density test is performed to determine the time distribution along the fine TDC bins. Then, the calibrated time that corresponds to the center of the bin is calculated for each bin from Equation (2).

(2) $$ti=\left[\frac{N_{i}}{2}+\sum_{k=0}^{i-1}N_{k}\right]\cdot \frac{T}{N}$$

where t*i* is the calibrated time of bin i, N is the total number of counts in the code density histogram, T is the delay of the fine TDC and N_i_ is the number of counts in the *i*th bin. It has been shown in a previous study [28,29] that the RMS errors are minimized when the bins are calibrated to the centers. In fact, the RMS error σ of the *b*th bin, when calibrated to a time t_c_, can be calculated from (3).

(3) $$\sigma^{2}=\frac{1}{t_{\max}-t_{\min}}\int_{t_{\min}}^{t_{\max}}{\left(t-t_{c}\right)}^{2}\;\mathrm{dt}=\frac{{\left(t_{\max}-t_{c}\right)}^{3}-{\left(t_{\min}-t_{c}\right)}^{3}}{3\left(t_{\max}-t_{\min}\right)}$$

where t_min_ and t_max_ are, respectively, the lower and upper time limits of this bin and t_c_ is the calibrated time of this bin (t_min_ < t_c_ < t_max_).

Considering that t_min_ = 0 and t_max_ = T_b_ (T_b_ is the bin width), Equation (3) can be written as:

(4) $$\sigma^{2}=\frac{{\left(T_{b}-t_{c}\right)}^{3}+t^{3}}{3T_{b}}={\;T}_{b}t_{c}{}^{2}-T_{b}{}^{2}t_{c}+\frac{T_{b}{}^{3}}{3}$$

The minimum RMS error is obtained when $t_{c}=\frac{T_{b}}{2}=\frac{\left(t_{\max}-t_{\min}\right)}{2}$, and the minimum RMS error is calculated from Equation (5).

(5) $$\sigma^{2}=\frac{{\left(t_{\max}-t_{\min}\right)}^{2}}{12}$$

**Figure 3:**
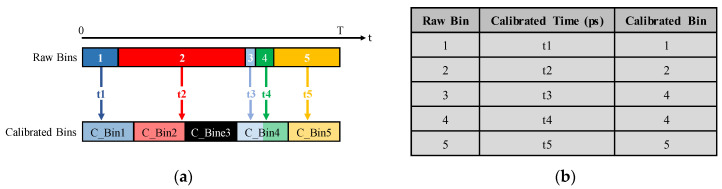
Bin-by-bin calibration: (**a**) Calculating the calibrated times and calibrated bins of the raw bins; (**b**) Bin-by-bin calibration lookup table.

The calibrated times of the raw bins calculated from Equation (2) are stored in a bin-to-time LUT that will be used later for the correction of the TDC’s non-linearity. Furthermore, the FSR of the TDC can be divided into calibrated bins with identical size. The calibrated time of the raw bins are then projected on the calibrated bins to determine which raw bin corresponds to which calibrated bin, as illustrated in Figure 3a. Thereafter, another LUT, namely the bin-to-calibrated_bin LUT, can be built to be used for the calibration of the measurement histogram or to convert the raw bin into a calibrated one in real-time, as presented in Figure 3b.

Figure 3a demonstrates that there are still large variations in the width of the calibrated bins. In this example, the third calibrated bin (C_Bin3) is a dead bin because the TDC has a large raw bin (Bin2). In addition, both the small raw bins (Bin3 and Bin4) are included in one corrected bin (C_Bin4).

#### 2.2.2. Average-Bin-Width Calibration Method

This method aims to divide the fine TDC into calibrated bins with identical widths. As for the bin-by-bin calibration, this method requires performing a code density. From this test, the delay of the time width of the *b*th raw bin (T_b_) can be calculated from Equation (6), where N is the number of counts of the code density histogram, N_b_ is the number of counts in the *b*th bin and T is the fine TDC’s delay:

(6) $$T_{b}=\frac{N_{b}}{N}$$

The idea of this calibration is to divide the fine TDC into M calibrated bins with identical time widths T_c_. Since T is the total delay of the fine TDC, T_c_ is calculated from Equation (7).

(7) $$T_{c}=\frac{T}{M}$$

Furthermore, since the calibrated bins have a uniform time width, these bins should contain the same number of counts N_c_, calculated from Equation (8), when performing a code density test:

(8) $$N_{c}=\frac{N}{M}$$

**Figure 4:**
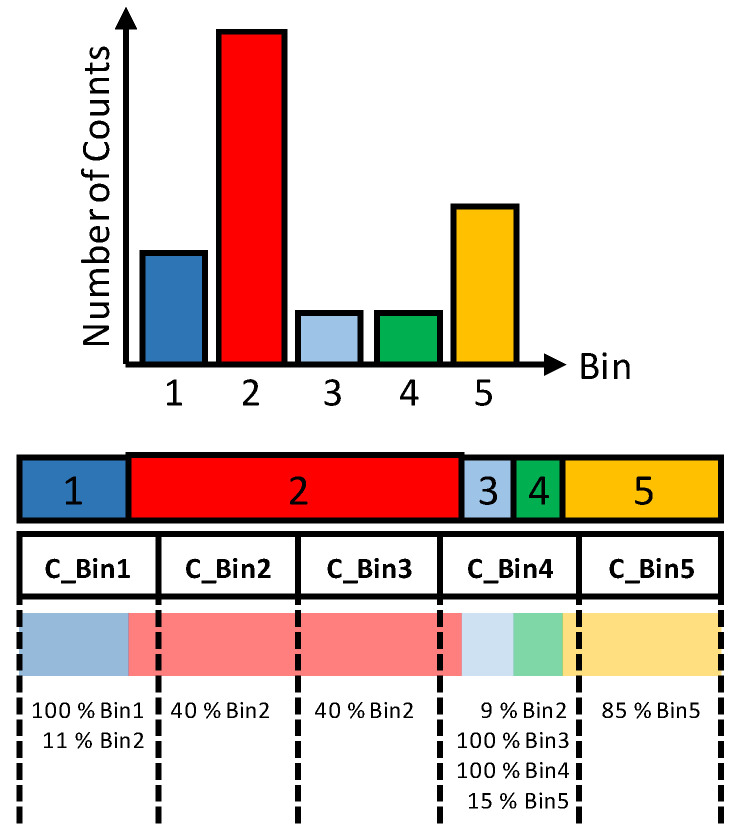
Average-bin-width calibration, redistribution of the total counts on identical calibrated bins and creating the calibration table that defines the percentage share of raw bins in each calibrated one.

**Figure 5:**
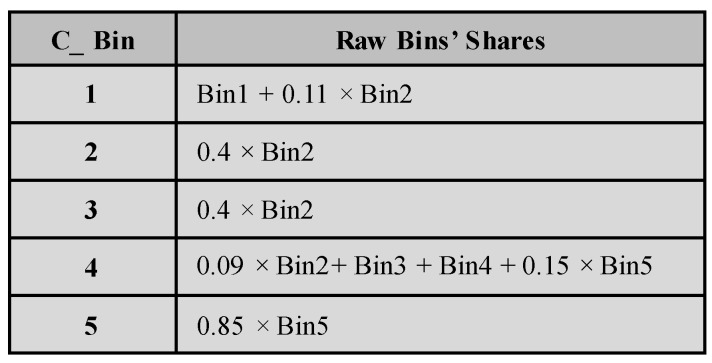
Calibration table of the fine TDC. It describes the shares of the raw bins in the calibrated bins.

Considering the code density histogram presented in Figure 2 that has five non-identical raw bins, in order to have five calibrated bins identical in size, the counts of the raw bins are successively redistributed on the calibrated bins starting from the first bin. For each calibrated bin, the percentage shares of the raw bins are calculated as demonstrated in Figure 4 and stored in a special table, named the calibration table, as presented in Figure 5. This table will be used later for the calibration of the measurement raw histogram.

Figure 4 illustrates that the calibrated histogram using this method has calibrated bins with identical size. This histogram has no dead bins because the large raw bin (Bin2) is distributed to four corrected bins (C_Bin1, C_Bin2, C_Bin3 and C_Bin4) with different percentages. It can also be noticed that the fourth corrected bin (C_Bin4) contains counts from four different raw bins (C_Bin2, C_Bin3, C_Bin4 and C_Bin5) also at different percentages.

Moreover, the TDC time resolution depends on the calibrated bin size; in other words, it depends on the number of calibrated bins. If the number of calibrated bins is L, the time resolution of the TDC after the calibration is calculated by Equation (9).

(9) $$T_{c}=\frac{T}{L}$$

## 3. Asynchronous TDCs Calibration

This section first explains the functionality of asynchronous TDCs as well as the bin-by-bin calibration for such TDCs. Then, it presents in detail our proposed methodology to calibrate asynchronous TDCs.

### 3.1. Operating Principle of Asynchronous TDCs

**Figure 6:**
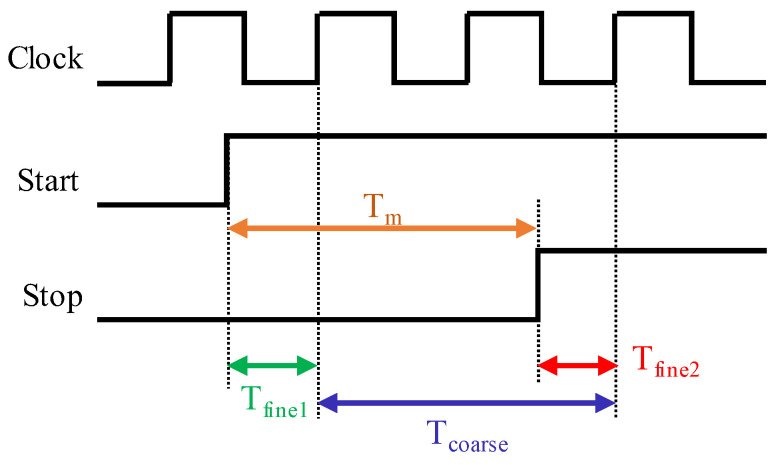
Asynchronous TDC chronogram. The time interval between two asynchronous signals to the system clock is calculated from three parts: two fine intervals and a coarse one.

In asynchronous TDCs, the Start signal is asynchronous with respect to the TDC clock, as is the Stop signal. Therefore, the time interval between these signals, Tm, is calculated using the following equations, as illustrated in Figure 6:

T_m_ = T_coarse_ + T_fine1_ − T_fine2_ (10)

T_m_ = Coarse × T_clk_ + T_fine1_ − T_fine2_ (11)

where T_fine1_ is the interval between the Start signal and the first rising edge of the clock that arrives after the Start signal, T_fine2_ is the interval between the Stop signal and the first rising edge of the clock after the Stop signal and T_coarse_ is the number of clock cycles between the mentioned clock rising edges multiplied by the clock period.

Hence, an asynchronous Coarse–Fine TDC should contain a coarse counter that measures T_coarse_ and two fine TDCs: one for the measurement of T_fine1_ called the Start fine TDC, and another one that measures T_fine2_ named the Stop fine TDC.

**Figure 7:**
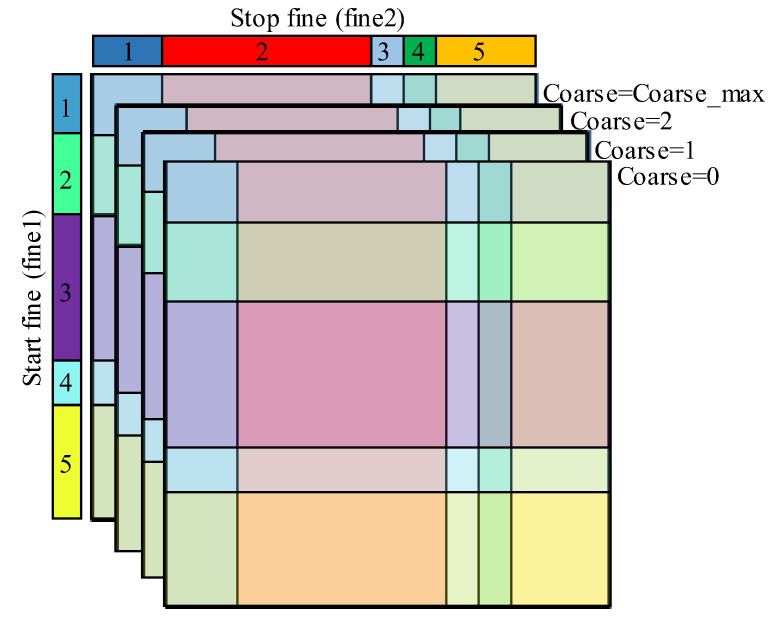
Three-dimension code density histogram of an asynchronous TDC with five-bin fine TDCs, the Stop and Start fine bin numbers are respectively represented on x and y, whereas the Coarse value is represented on z.

It is evident from Equation (11) that, in asynchronous TDCs, each count can be represented by three values: Start fine bin number (fine1), Stop fine bin number (fine2) and the coarse counter value (coarse). Therefore, the measured counts can be compiled in a 3-D histogram. Again, to illustrate the purpose, let us consider a 3-D asynchronous TDC with five bins for the Start and Stop fine TDCs and a clock period T. Figure 7 illustrates a 3-D code density histogram of such a TDC.

### 3.2. Calibration Methods for Asynchronous TDCs

#### 3.2.1. Bin-by-Bin Method

**Figure 8:**
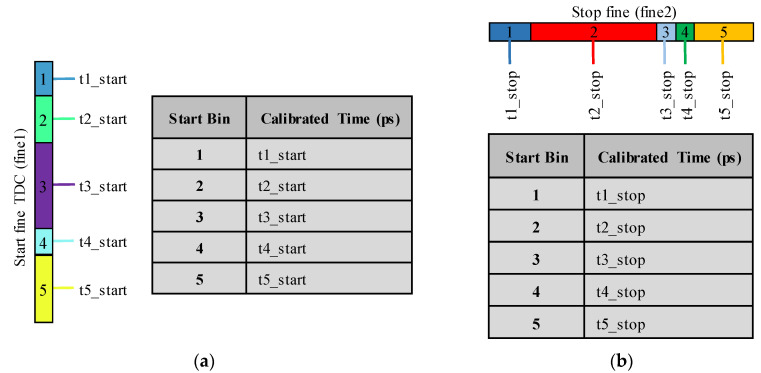
Lookup tables of the two fine TDCs: (**a**) Start fine TDC LUT; (**b**) Stop fine TDL LUT.

To calibrate an asynchronous TDC using the bin-by-bin method, firstly, a code density test is performed to calculate the calibrated times of the raw bins and build the lookup tables of the two fine TDCs, as explained for synchronous TDCs in II-B-1. Figure 8 shows the built LUTs.

Thereafter, the calibrated interval of each cell of the 3-D histogram can be calculated, using the lookup tables built in the previous step, from the following equation:

t_cell = (Coarse × T) + t_start − t_stop(12)

where t_cell is the calibrated time of the cell, t_stop, t_start are the calibrated times of the Stop and Start bins that represent the (x, y) coordinates of the cell, T is the system clock period and Coarse is the cell coarse value.

The calibrated intervals of all the cells are then saved in a 3-D LUT that can be used for the correction of the TDC’s non-linearity. Furthermore, the FSR of the TDC can be divided into calibrated bins with identical size to determine to which calibrated bin, in the final calibrated 1-D histogram, corresponds each cell of the 3-D raw histogram, according to its calibrated interval. Finally, a cell-to-calibrated_bin 3-D LUT can be built to be used later for the calibration of the measurement to convert its 3-D raw histogram to a 1-D calibrated one.

#### 3.2.2. Matrix Calibration

Matrix calibration is based on the average-bin-width method and used for the calibration of asynchronous Coarse–Fine TDCs. It requires performing a code density test with a sufficient number of counts for which the Start and the Stop signals arrive at different delays, asynchronously to the system clock, in a way that they cover the FSR of the Start and Stop fine TDCs. Considering the 3-D code density histogram, illustrated in Figure 7, each cell stores the number of counts for which the Start and Stop signals arrive in the bins that respectively correspond to the x and y coordinates of this cell and with a coarse value equal to its z coordinate. For instance, the cell (4, 3, 1) saves the number of counts in which the Start signal arrives in the fourth bin of the Start fine TDC, and the Stop signal arrives in the third bin and with a coarse value equal to 1. In the case of ideal Start and Stop fine TDCs with identical raw bins, all the cells of the code density histogram would have an identical size. In a real TDC, since the fine TDCs have non-uniform raw bins, the cells of the 3-D code density histogram have different sizes. The size of each cell, represented by its number of counts, depends on the width of its Start and Stop raw bins.

One way to calibrate the 3-D code density histogram is to make all the cells have a uniform size. The matrix calibration consists in redistributing the code density counts evenly on calibrated cells identical in size. This can be achieved in four steps:

1. Step 1: Individual calibration of the Start and Stop fine.

**Figure 9:**
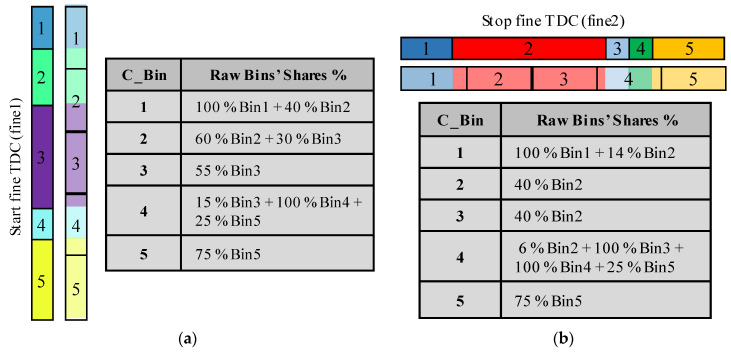
Individual calibration tables: (**a**) Start fine TDC calibration table; (**b**) Stop fine TDC calibration table.

In fact, if the Stop fine and the coarse values of the 3-D code density histogram cells are ignored, the columns will be merged in one column. This column is a 1-D histogram that represents a code density histogram of the Start fine TDC. Likewise, merging all the rows, by ignoring the Start fine and the coarse values, provides a 1-D code density histogram of the Stop fine TDC. These histograms can be used to build the calibration table of the Start and Stop fine TDCs, as described in II-B-2 for the average-bin-calibration of synchronous TDCs. Figure 9 shows the built calibration tables.

2. Step 2: Column calibration.

**Figure 10:**
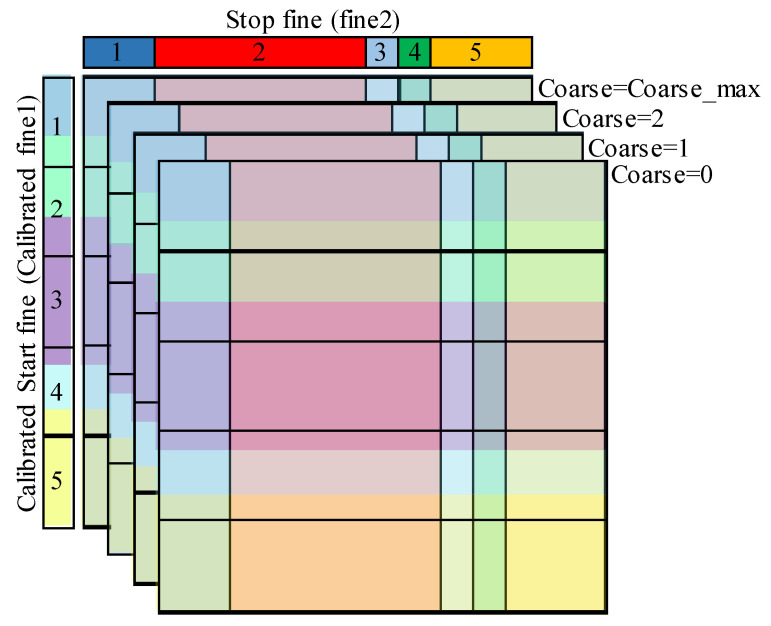
Column calibration, the individual calibration of the columns using the average-bin-width method gives a semi-calibrated histogram where all the rows have the same height.

In practice, each column of the 3-D code density histogram can be considered a 1-D code density histogram of the Start fine TDC, and thus can be calibrated using the Start calibration table built in the previous step. The individual calibration of all the columns of the 3-D histogram results in a semi-calibrated 3-D histogram in which all the cells have the same row height while the columns still have non-uniform widths, as illustrated in Figure 10.

3. Step 3: Row calibration.

**Figure 11:**
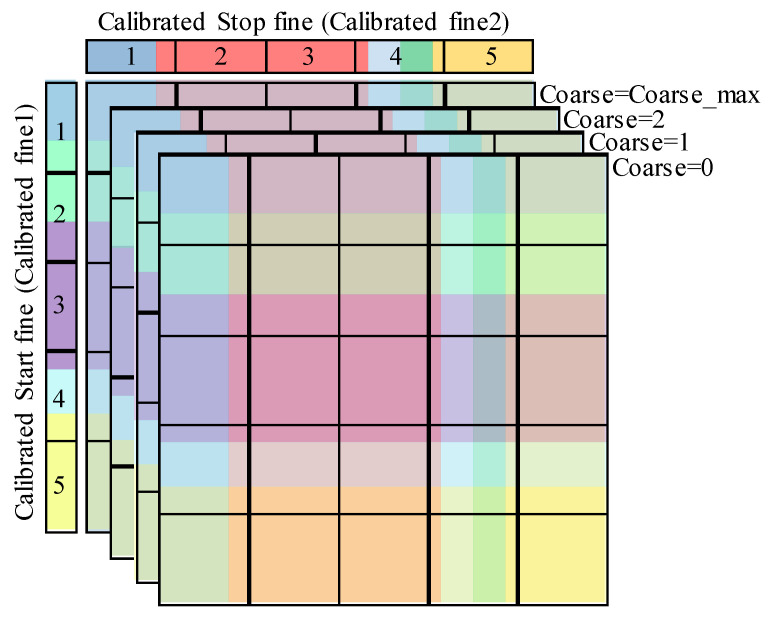
Row calibration; the average-bin-width calibration is applied to the rows, resulting in a calibrated 3-D histogram with identical cell size.

The rows of the semi-calibrated histogram resulting from the previous step are practically 1-D code density histograms of the Stop fine TDC. Hence, they can be calibrated using the calibration table of this TDC. The individual calibration of all the rows provides the calibrated 3-D histogram where all the cells have identical size, as shown in Figure 11.

4. Step 4: Building 1-D calibrated histogram.

The last step of the matrix calibration aims to convert the 3-D histogram resulted from the previous step into a 1-D histogram. In fact, each cell of the 3-D calibrated histogram should be added to its corresponding bin in the 1-D calibrated histogram. The number of this bin is calculated by Equation (13).

Bin_number = (Coarse × M) + C_fine1 − C_fine2(13)

where C_fine1, C_fine2, Coarse are, respectively, the x, y, z coordinates of the calibrated cell, i.e., the numbers of its row, column and slice, and M is the total number of calibrated bins in the Stop fine TDC, which equals 5 in our example. For instance, the counts of the cell (3, 2, 4) is part of the 21st bin of the calibrated 1-D histogram, since M = 5 and, thus, the number of the bin to which corresponds this cell is ((4 × 5) + 3 − 2 = 21).

Consequently, in this step, all the cells of the 3-D calibrated histogram should be scanned and added to their corresponding bins in the 1-D calibrated histogram.

## 4. Simulation Results

Different simulations were carried out using MATLAB to compare the studied calibration methods for synchronous and asynchronous TDCs. The simulated TDCs are based on the Nutt method and consist of fine and coarse TDCs. The coarse TDC is a simple counter and the fine TDC is a TDL with 256 delay elements. The total delay of the TDL is 5 ns distributed on the delay elements with the same profile of the time distribution along a real TDL implemented on a Cyclone V FPGA [11].

### 4.1. Synchronous TDCs

**Figure 12:**
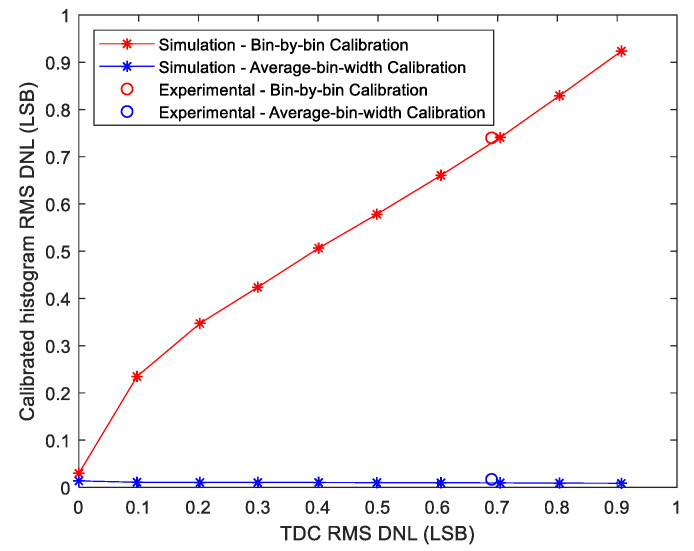
Simulation and experimental results for synchronous TDCs: the DNL after applying the calibration methods compared to the DNL of the raw TDC.

In the first simulation, 10 synchronous TDCs were simulated with different Root Mean Square (RMS) differential non-linearity (DNL) that varied from 0 to 1 LSB. For each simulated TDC, 10^7^ random events were simulated to perform a code density. The Stop signal for these events arrived with random delays uniformly distributed over the total delay of the TDC’s TDL. The resulting code density histogram was used to build the calibration tables of the average-bin-width calibration and the LUTs of the bin-by-bin method. Thereafter, for the evaluation and comparison of these two methods, another code density test, was performed with another 10^7^ simulated random events. Then, the two methods were applied to calibrate the resulting code density histogram. After repeating these steps for each of the 10 simulated TDCs, the RMS DNL of the calibrated histograms was calculated to evaluate the calibration method. Figure 12 illustrates the obtained results and shows that the bin-by-bin method did not improve the DNL of the TDC, whereas the average-bin-width calibration was independent to the noise of the TDC and significantly improved the DNL.

It should be pointed out that since the DNL of the first TDC in this simulation was 0 LSB, i.e., an ideal TDC, the DNL after applying the calibration should theoretically be 0 LSB. Nevertheless, the calibrated histogram had a DNL of about 0.005 LSB. This is because code density tests are limited by the shot noise, which can be calculated from Equation (14).

(14) $$\mathrm{Shot}\;\mathrm{noise}=\sqrt{\frac{\mathrm{Number}\;\mathrm{of}\;\mathrm{Bins}}{\mathrm{Counts}\;\mathrm{number}}}$$

In our case, the number of bins was 256 and the counts number was 10^7^, and this equation gives about 0.005 LSB.

**Figure 13:**
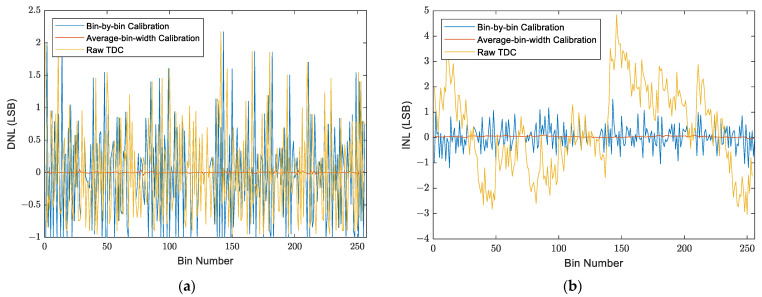
DNL and INL values for a synchronous TDC, before and after applying the two calibration methods: (**a**) DNL values; (**b**) INL values.

The second simulation aimed to compare the DNL and the integral non-linearity (INL) of the two calibration methods when applied to a simulated TDC that has the same time distribution as a real one implemented on a Cyclone V SoC-FPGA [11]. As for the first simulation, two code density tests, with 10^7^ events each, were simulated. The first test was to build the bin-by-bin LUT and the average-bin-width calibration table. The second test was to apply the two calibration methods and to compare between them. Figure 13 presents the DNL and INL values of the calibrated histograms obtained after applying the two methods as well as those of the non-calibrated histogram, and Table 1 summarizes the data statistics of these values. The obtained results show that the bin-by-bin calibration improved just the INL of the TDC without improving its DNL, whereas the average-bin-width calibration significantly improved both the DNL and the INL.

**Figure 14:**
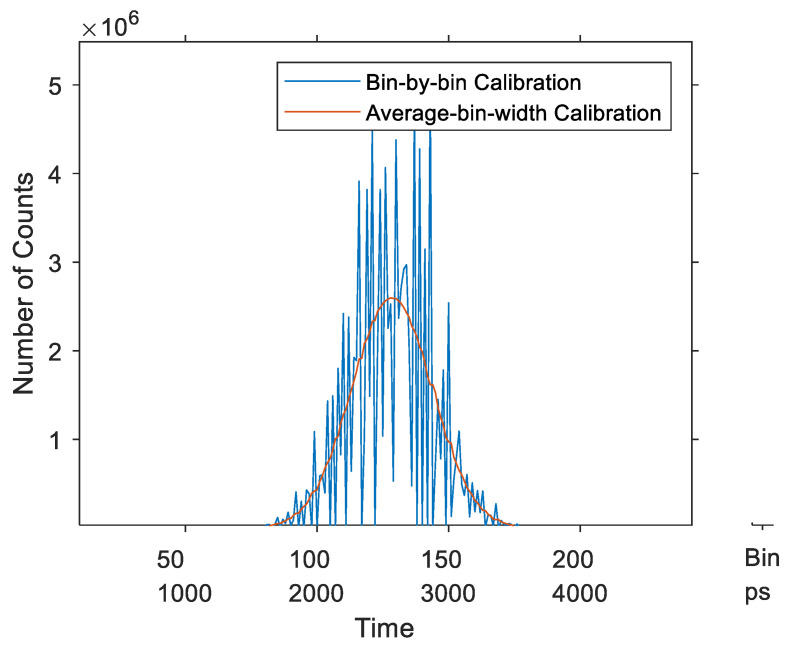
Calibrated histogram of a Gaussian signal—synchronous TDCs.

The third simulation demonstrates the advantage of the average-bin-width over the bin-by-bin calibration applied to histograms. In this simulation, a Gaussian signal was measured by a simulated TDC that had the time distribution of the real TDC, as in the previous simulation. Ten million (10^7^) events were simulated following a normal distribution with an arbitrary chosen average delay of 2.5 ns and standard deviation (sigma) of 0.3 ns. The two calibration methods were then applied to calibrate the recorded histogram. Figure 14 shows the resulting calibrated histograms of the Gaussian signal. It is evident that the average-bin-width calibration had much less noise than the bin-by-bin method.

### 4.2. Asynchronous TDCs

**Figure 15:**
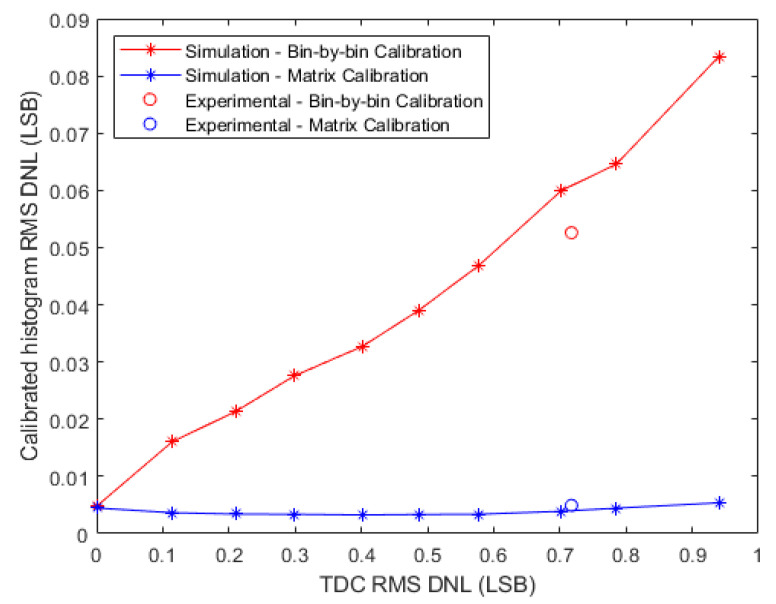
Simulation and experimental results for asynchronous TDCs: the DNL after applying the calibration methods compared to the DNL of the raw TDC.

In the first simulation, 10 asynchronous TDCs were simulated with RMS DNL values that varied between 0 LSB and 1 LSB. These DNL values were measured after concatenating the raw bins of the two fine TDCs of each asynchronous TDC. Thereafter, for each simulated TDC, a code density test was simulated with 10^7^ random events to build the bin-by-bin LUTs and the calibration tables of the matrix calibration. Then, for the evaluation and the comparison between the two methods, another code density test was simulated with 10^7^ events. For these events, the Start signal arrived with random delays uniformly distributed over the range of the Start fine TDC, and the Stop signal arrived after the Start signal by time intervals that varied uniformly from 0 to 5 ns. From the arrival times of these events, a 3-D raw histogram was built by calculating the coordinates of each event, i.e., its Start fine bin, Stop fine bin and coarse value, and incrementing the corresponding cell by one. Thereafter, the bin-by-bin and the matrix calibration methods were applied to calibrate the 3-D raw histograms and deduce 1-D calibrated histograms. The RMS DNL values of these calibrated histograms were measured to compare the calibration methods, and the results are presented in Figure 15.

The results illustrated in Figure 15 show that the DNL values of the calibrated histograms obtained by the bin-by-bin method were improved by a factor of 10 and linearly increased with the DNL of the TDC. In contrast, the proposed matrix calibration method was much less sensitive to the noise of the raw TDC with an almost flat response. The error of the ideal TDC (noise 0 LSB) is also due to the shot noise, as discussed for the synchronous TDC, and can be calculated by Equation (14) and equals 0.005 LSB.

In the next simulation, an asynchronous TDC was simulated with 256-delay-element Start and Stop fine TDCs that had the same time distribution as an asynchronous TDC implemented on a Cyclone V FPGA [11]. Firstly, a code density test was simulated to build the LUTs and the calibration tables in the same way as in the previous simulation. Then, the bin-by-bin and the matrix calibration methods were applied to calibrate the raw histogram of another simulated code density test. Thereafter, for the evaluation of these two methods, the obtained calibrated histograms were compared with the non-calibrated one in terms of the DNL and INL values.

**Figure 16:**
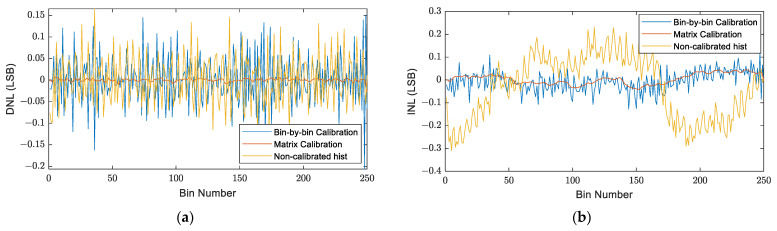
DNL and INL values, for an asynchronous TDC, before and after applying the two calibration methods: (**a**) DNL values; (**b**) INL values.

Figure 16 shows the DNL and INL values after applying the calibration methods as well as those of the non-calibrated histogram. Table 2 summarizes the data statistics of these values.

The obtained results show that the bin-by-bin method improved the INL, compared with the non-calibrated histogram, without improving the DNL. However, the matrix calibration is more than 10 times better than the bin-by-bin method in terms of the DNL and about 2 times better in terms of the INL. Nevertheless, comparing these results with those obtained for a synchronous TDC, presented above in Figure 13 and Table 1, it can be noticed that the DNL and INL of the non-calibrated histogram of an asynchronous TDC are about 10 times less accurate than their values for a synchronous TDC.

**Figure 17:**
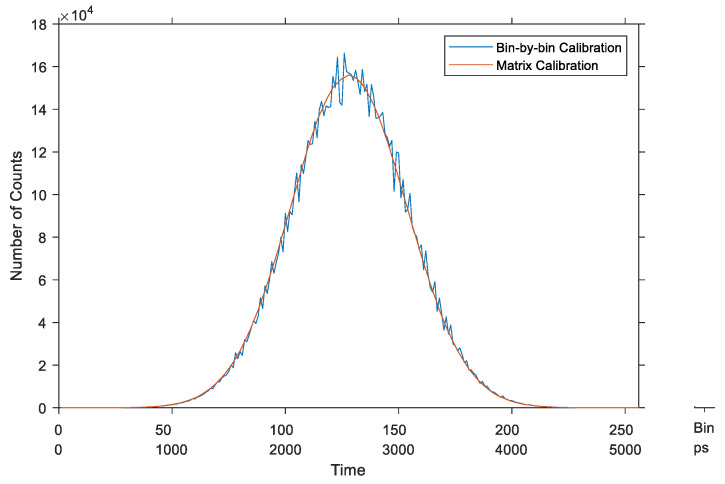
Calibrated histogram of a Gaussian signal—asynchronous TDCs.

The last simulation compared the two methods applying each to a calibrated measurement histogram of Gaussian signal. Using the simulated TDC of the previous simulation, a Gaussian signal was simulated by 10^7^ events. The time interval between the Start and Stop signals of these events followed a normal distribution with arbitrary chosen average delay and standard deviation of 2.5 ns and 0.5 ns, respectively. The obtained results depicted in Figure 17 show that the average-bin-width calibration had less noise than the bin-by-bin method. Furthermore, the center of gravity of the calibrated histogram was 2489.5 ps (error = 10.5 ps) for the bin-by-bin method, whereas it was 2499.1 ps (error = 0.9 ps) for the matrix calibration.

## 5. Experimental Results

In this section, experiments were performed on real TDCs, implemented on a Cyclone V FPGA, to confirm the simulation results for synchronous and asynchronous TDCs.

### 5.1. Synchronous TDCs

In this experiment, a synchronous TDC was implemented on the FPGA kit. The coarse TDC was an 8-bit counter and the fine TDC was a Tapped Delay Line (TDL) of 256 delay elements. The system clock period was 5 ns, i.e., the fine TDC range was 5 ns. The Start signal was synchronized with the system clock, whereas the Stop signal was connected to the output signal of a Single-Photon Avalanche Diode (SPAD) to build the LUT and the calibration table of the bin-by-bin and the average-bin-width calibration methods, respectively. A code density test with about 10^7^ events was performed by exposing the SPAD to the ambient light at a low detected photon rate of about 1 M photon/s. At such a relatively low photon rate, the mean time between the arrival of two successive photons is 1 µs, which is 200 times larger than the total delay of the fine TDC. Thus, the Stop signal arrived with random delays that covered the FSR of the TDC. From the code density histogram, the RMS DNL of the raw TDC was calculated and it was about 0.69 LSB. Thereafter, another code density test was performed with the same number of events to evaluate the calibration methods. The code density histogram was then calibrated using the two methods and the RMS DNL was calculated for the resulting calibrated histograms. For the bin-by-bin method, the RMS DNL was 0.74 LSB, whereas it was just 0.017 LSB for the average-bin-width method. These experimental values confirm the simulation results, as plotted in Figure 12.

**Figure 18:**
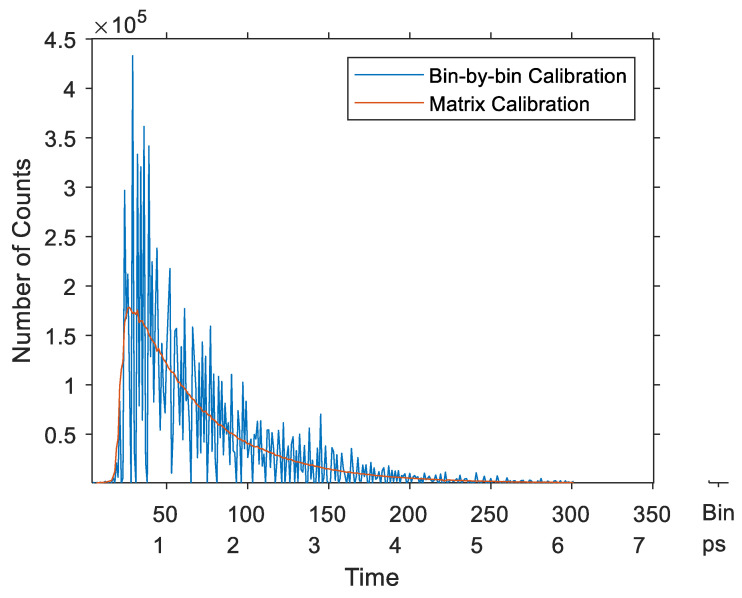
Fluorescence signal of a piece of paper using the two calibration methods—synchronous TDC.

Moreover, the implemented system was used in real conditions to record the fluorescence signal of a piece of paper excited by a 405 nm laser pulse [30]. Figure 18 shows the calibrated histogram of the recorded signal using the two calibration methods. It confirms that the average-bin-width calibration had much less noise than the bin-by-bin method.

### 5.2. Asynchronous TDCs

To experimentally verify our proposed method “the Matrix Calibration” and to confirm the simulation results, an asynchronous TDC was implemented on the FPGA kit. This TDC consisted of an 8-bit coarse counter, clocked at a system clock frequency of 200 MHz, and two TDL-based fine TDCs with 256 delay elements each. In addition, a separate on-chip PLL generated an asynchronous Start signal, whereas the Stop signal was connected to the output signal of a SPAD. Firstly, a code density test was performed by exposing the SPAD to the ambient light at a low detected photon rate of 1 M photon/s. Therefore, the arrival time of the Start and Stop signals of the measured events can be considered uniformly distributed over the range of the fine TDCs. From the code density histogram, the 3-D LUT and the calibration tables were built for the bin-by-bin and the matrix calibration, and the RMS DNL of the fine TDCs was calculated (0.71 LSB). Thereafter, another code density test was performed and the resulting histogram was calibrated by the two calibration methods. The RMS DNL of the resulting calibrated histograms were calculated and the results are as following: (0.053 LSB) for the bin-by-bin method and only (0.005 LSB) for the matrix calibration, as plotted on Figure 15. Indeed, in order to have experimental results comparable with the simulation, the same number of events must be measured in the experimental code density tests as in simulation, i.e., 10^7^ events.

**Figure 19:**
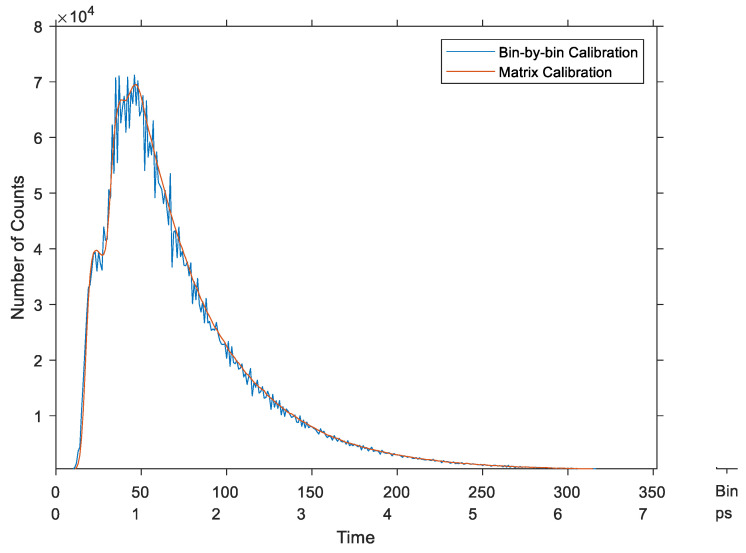
Fluorescence signal of a piece of paper using the two calibration methods—Asynchronous TDC.

As for the synchronous TDC, in the last experiment, the implemented asynchronous TDC was used in real conditions to record the fluorescence signal of a piece of paper. Figure 19 shows the calibrated histogram of the recorded signal obtained after applying the bin-by-bin and the matrix calibration methods. This figure shows that the matrix calibration had less noise than the average-bin-width method. Nevertheless, the bin-by-bin calibration, when applied to asynchronous TDCs, has much less noise than when it is applied to synchronous ones.

### 5.3. Processing Speed Comparison

The average-bin-width and the matrix calibration methods lead to better results in terms of noise than the bin-by-bin method. The drawback is a more complex signal processing which includes more multiplications and data access. As mentioned before, the average-bin-width calibration and the matrix calibration are post-processing on the TDC raw data. Moreover, the bin-by-bin method for asynchronous TDCs is very complicated to implement for online calibration, and it is easier to be performed as post-processing. Thus, all the studied calibration methods were implemented as post-processing. The SoC-FPGA kit used in experiments integrates a hard processor system (HPS), namely the ARM Cortex A9 processor, with a Cyclone V FPGA fabric and provides a high-speed interface for the data transfer between these two parts. The TDC systems were implemented on the FPGA and the data were transferred into the SDRAM of the HPS to be processed by performing the different calibration techniques and other data processing. A set of experiments were carried out to compare the speed of the different calibration methods on the implemented TDCs, knowing that the number of raw bins in a fine TDC is 256 bins.

Table 3 compares the processing time between the bin-by-bin calibration for asynchronous TDCs and the matrix calibration. It compares the time of applying the calibration without considering the time of creating the LUTs and the calibration tables. This table shows that the speed of the bin-by-bin calibration depends only on the maximum value of Coarse (Coarse_max), because the size of the 3-D LUT is always equal to (the number of raw bins in Start fine TDC × number of raw bins in Stop fine TDC × Coarse_max), as illustrated in Figure 7, whereas the matrix calibration speed depends on the total number of calibrated bins in the histogram, as shown in Figure 5. Furthermore, the ratio between the speed of the two methods has almost a linear relationship with the number of calibrated bins in the clock period; this ratio has a maximum value of about 8 when the number of calibrated bins in a period is equal to the number of raw bins.

The same experiments were performed on a synchronous TDC. The obtained results show that the ratio between the speed of the average-bin-width has an almost linear relationship with the number of calibrated bins in clock period; the maximum value of this ratio is about 4 when the number of calibrated bins in a clock period is equal to the number of raw bins of the fine TDC.

## 6. Conclusions

This paper covers in detail the methodology of the most commonly used calibration methods for synchronous and asynchronous TDCs, which are the bin-by-bin and average-bin-width methods. It also introduces a novel calibration method for asynchronous TDCs called the “Matrix calibration”. Simulations and experiments were carried out to compare these methods. The results show that, for synchronous TDCs, the average-bin-width calibration is much better than the bin-by-bin method, which does not improve the DNL of the raw TDC. The obtained results also affirm that the proposed method for asynchronous TDCs is less sensitive to the DNL of the raw TDC and is up to 10 times better than the bin-by-bin method applied for histogram measurements. This improvement occurs at the expense of a longer calibration time due to the complexity of the signal processing that includes more multiplication and memory access instructions. Furthermore, experimental results obtained for real TDCs, implemented on Cyclone V FPGA, confirmed the simulation results. However, it should be pointed out that the proposed calibration method does not improve the TDC precision in the case of single-shot measurements.

The proposed method has been effectively applied in a Time-Correlated Single Photon Counting (TCSPC) system including asynchronous TDCs. Another paper about this system will be published in the future. Furthermore, this method can be extended to be applied for the calibration of other systems dealing with multidimensional histograms involving more than three dimensions.

## Figures and Tables

**Table 1:** DNL and INL statistics for a synchronous TDC calculated for the non-calibrated histogram and the calibrated histograms of the two methods.

		Mean	Median	STD
DNL data statistics	Raw TDC	0	−0.1054	0.7362
	Bin-by-bin	0	−0.07453	0.7798
	Average-bin-width	0	−0.0003624	0.009665
INL data statistics	Raw TDC	0.2614	0.2038	1.676
	Bin-by-bin	0.003083	0.007194	0.4816
	Average-bin-width	0.0026	0.006688	0.03167

**Table 2:** DNL and INL statistics for an asynchronous TDC calculated for the non-calibrated histogram and the calibrated histograms of the two methods.

		Mean	Median	STD
DNL data statistics	Non-calibrated histogram	~0	−0.0049	0.056
	Bin-by-bin calibration	~0	−0.0036	0.058
	Matrix calibration	~0	0.00033	0.0035
INL data statistics	Non-calibrated histogram	−0.034	−0.008	0.14
	Bin-by-bin calibration	−0.0064	−0.004	0.045
	Matrix calibration	0.0036	0.0048	0.023

**Table 3:** Calibration processing speed comparison between the bin-by-bin method for asynchronous TDCs and the matrix calibration.

Total Number of Bins in Calibrated Histogram	Number of Calibrated Bins in Clock Period	Maximum Value of COARSE	Calibration Process Speed (CPU Tick)		Ratio (Matrix/Bin-by-Bin)
			Bin-by-Bin Calibration	Matrix Calibration	
4609	256	19	543,2528	42,229,555	7.77
1537	256	7	220,7046	18,058,416	8.18
513	256	3	1,197,789	9,453,249	7.89
385	256	2	952,921	7,493,491	7.86
301	200	2	959,247	5,279,525	5.5
226	150	2	936,000	3,681,132	3.93
151	100	2	938,161	2,501,956	2.67
76	50	2	930,375	1,467,180	1.58

## Data Availability

Not applicable.
